# Nitrous Oxide Gas

**Published:** 1881-07

**Authors:** Laurence Turnbull

**Affiliations:** Philadelphia


ARTICLE II.

Continued from page 232.

### NITROUS OXIDE GAS.

BY DR. L. TURNBULL, PHILADELPHIA.

Our reviewer dwells on the difficulty of the preparation of nitrous oxide, and the impossibility of transportation, unless with costly apparatus, thus preventing its use everywhere, except in hospitals, and it is not adapted for general surgical practice. He concludes what he has to say of it by reporting the following case: "It occurred to Nussbaum. The patient was a hard drinker, upon whom perineal section had been performed. For the passage of a bougie he had taken chloroform fifty-three times; the fifty-fourth time he took nitrous oxide and never woke up. Deep cyanosis came on during the administration, and he died, notwithstanding respiration continued for fifty minutes, and was maintained fifteen minutes longer by faradization of the phrenic nerve." We are not informed whether the simple operation of bringing forward the jaw or pulling out and holding the tongue from falling back, had been employed, or most important of all any examination of his kidneys. Our reviewer further states "Especially adapted as is nitrous oxide for dental operations, and safe as it is has been shown to be, the administration of any other anæsthetic by a dentist should be considered criminal"! the exclamation point is ours. Why not also the general surgeon for using chloroform in trifling operations; now, we do not consider nitrous oxide gas difficult to make; we have often made it, and almost any medical student after his first year at chemistry can make it. Besides, it is now made and sold in liquid form, and at a very reasonable rate, with all the necessary apparatus (see p. 197 of our work), and so one of the great drawbacks to its extended use has been done away with, as in this form it can be kept in iron cylinders for any length of time, and transported to any part of the country. When wanted the necessary quantity is allowed to pass into the rubber bag, carefully avoiding the introduction of water or atmosphere air or nitric oxide fumes, which is sometimes found as an impurity.

We avail ourselves of the work of "Rottenstein"* and Jones'† report for any new facts on the subject of nitrous oxide, not contained in our own work. Dr. Rottenstein gives the ordinary historical statement of the idea of Sir H. Davy, (1799), that owing to the property of destroying pain, nitrous oxide gas might be employed with advantage in surgical operations. The experiments of Davy on himself are given in detail, followed by those by Berzelius, Vanqelin, Thenard, Orfela and others. "The gas," as Dr. Rottenstein remarks, "was an object of curiosity, and given for amusement until 1844, when Horace Wells made the remarkable discovery which immortalized him." He then summarizes the history of 'Well's' experiments, but fails to state that when he attempted to administer the gas in an extensive surgical operation in Boston, he failed by employing a too small quantity, and it was not until April, 1848, that Dr. Bigelow, of Boston, demonstrated its value in surgery by performing an excision of the breast, using sixty gallons of the gas. But at the present day nitrous oxide has had its proper place assigned to it, that of short operations in surgery or in conjunction with other anæsthetics as chloroform, ether, ethydine dichloride, etc. There has not occurred a single death in Philadelphia from nitrous oxide. I have reported but four cases of death in which it took a part, and although not the primary cause in our estimation, in one or two cases, from being impure or not fresh, or improperly employed, it exerted an unfavorable action upon the patient, and like every other anæsthetic will cause death at the times or very unfavorable symptoms, requiring a careful and judicious administration and the proper selection of cases for its use.

Dr. Rottenstein passes over in review the experiments and deductions of Krishaber, Hermann, Jolyet, Blanche, Zuntz, Golstein, Paul Bert, on the physiological action and clinical application of the protoxide of nitrogen. Hermann, with a mixture of oxygen and nitrous oxide (one volume to four), besides, the other effects, noticed with the diminution of sensibility to pain that the tactile sensibility was preserved for a considerable time; there was a state of excitement, consciousness was never completely lost, and anæsthesia was never complete; if deprived of the oxygen by breathing into a spirometer, dysp-

*"Traite D'Anæsthesia Chirurgical."

†Report on Anæsthetics by H. Macnaughton Jones, M. D., Pamphlet, p. 51, Dublin, 1881.

nœa quickly supervened. On the other hand, with the pure gas, all the symptoms and consequences of asphyxia were manifest. The admission of air dissipated these symptoms. Hermann concludes that the elementary phenomena resulting from the use of the protoxide are not other than those produced by indifferent gases; as with hydrogen, on agitating arterial blood with the gas, it becomes black; venous did not become arterial, the co-efficient absorption of blood for this gas ($N_2O$) is scarcely equal to that of distilled water. Its fatal action on the heart of the frog is manifested more quickly than in the case of hydrogen. Its elimination is experimentally demonstrated to take place through the lungs (Traite d'Anesthesia, p. 85). Krishaber's experiments were directed to solve these four questions: (1) If protoxide of nitrogen produces complete insensibility? (2) Will it cause death like other anæsthetics, and to what are we to attribute the death? (3) Has it advantages over other anæsthetics? (4) In what class of cases is it specially indicated? He proceeded by a series of experiments on rabbits with the protoxide gas mixed with air; with the gas in its pure state, with chloroform, and by comparing these effects with those produced by depriving them of air—in other words, by asphyxiating them—this latter process being effected by ligaturing the trachea. When giving the gas pure, the complete exclusion of atmospheric air was secured by laying bare the trachea, performing tracheotomy, inserting a silver tube, which, when the animal had breathed naturally through it for a few minutes, was connected with a balloon of nitrous oxide gas, and then the trachea was ligatured above (vid. pp. 86-93) Conclusions*—protoxide of nitrogen brings about anæsthesia and death in the same manner as chloroform; simple asphyxia does not produce insensibility, and its effects on the heart and respiration are very different from those of anæsthetics; the nitrous oxide gas is distinct from chloroform in the rapid succession of symptoms, not progressive and regular, early disturbing the heart's rhythm and producing irregularity, and the anæsthesia, rapid in its appearance, disappears as quickly; thus the rapid fatality of the gas counterbalances the advantages derived from its fugitive character. The experiments of Jolyet and Blanche we have given in full in our work; they were conducted with the view of

*The conclusions of Krishaber are not in accord with the received opinions of the day.

determining the action of nitrous oxide on the respiration. They found the gas arrested the germination of plants and equally, in varying intervals, the respiration of different animals, and that the anæsthesia was due to the deprivation of the blood of oxygen, but mixed with oxygen there was no anæsthesia. Goldstein conducted his experiments in the laboratory of Pflüger, at Bonn, with the assistance of Professor Zuntz. They failed to arrive definitely at a conclusion as to whether the small quantity of oxygen found during the decomposition of the protoxide of nitrogen was utilized in the system, but they proved that not alone the blood but the other organic liquids of the body absorb the gas. Frogs respiring pure nitrous oxide gas lost all reflex excitability at the end of five minutes, whereas with hydrogen the reflex excitability continued for a quarter of an hour. Golstein concluded that the absence of oxygen was an essential in the action of the nitrous oxide, and the rapid recovery on the admission of a small quantity of air shows how the interruption of asphyxia arrests that action. He distinguishes three phases in the asphyxia of the nitrous oxide; in the first the respiratory acts are diminished, and this stage corresponds to the first phase of ordinary asphyxia, whereas, in the second phase, active breathing ceases much more quickly than in ordinary asphyxia (65 ($N_2O$) to 102–108 seconds —asphyxia); sensibility also is lost in the second phase of nitrous oxide asphyxia; it is preserved in ordinary asphyxia; hence the advantage of the former—anæsthesia being already obtained in this second phase, it is completely useless for the practitioner to continue narcotization.

M. Paul Bert ("Mémoire lu à l'Académie des Sciences le, 11 November, 1878,") attracted by the difficulty of administering nitrous oxide gas without the admission of air; and hence the simultaneous occurrence of asphyxia, and anæsthesia, endeavored to prolong indefinitely the anæsthesia, and so utilize the gas for lengthy operations, starting with the knowledge that the tension of the gas (pure) must be equal to one atmosphere, in order that it may diffuse in sufficient quantity through the organism—that is, under normal pressure, cent. per cent., by placing the patient so that the pressure could be increased to two atmospheres—he conceived the idea that we might obtain the desired degree of tension while admitting fifty per cent. of air, thus maintaining in the blood the normal quantity of oxygen and permitting of the respiratory act. A mixture of five-sixths of protoxide and one-sixth of oxygen gives exactly a tension equal to one atmosphere. Placing in a chamber, under the increase of pressure of one-fifth of an atmosphere, a dog, and making him breathe the above mixture, the animal, after a few minutes, was completely anæsthetized, evidenced by complete insensibility, continuing for an hour without change. The blood preserved its red color, the heart-beats their force, the temperature its normal degree, and reflex excitability was maintained; recovery occurred after a few free respirations of air; so far as sensibility, will, intelligence, the animal regained its vivacity, and when liberated walked. This rapid recovery, M. Paul Bert says, indicates that, not as in the case of chloroform, the nitrous oxide is simply dissolved in the blood, escapes quickly by the lungs, and does not form any chemical combination; in destroying the nervous sensibility it does not prevent the reflex acts of organic life, while the ready return to the normal state, when life is endangered, through the free admission of air.

(TO BE CONTINUED.)

